# Immunological Responses and Protection in the Largemouth Bass (*Microterus salmoides*) Immunized with Inactivated Vaccine Against Largemouth Bass Ranavirus (LMBRaV)

**DOI:** 10.3390/ani15060803

**Published:** 2025-03-12

**Authors:** Tao Yang, Jiale Zhai, Chenyang Li, Lingbing Zeng, Yiqun Li, Wenzhi Liu, Yan Meng, Yuding Fan, Zhenyu Huang, Yong Zhou, Nan Jiang

**Affiliations:** 1 Department of Aquatic Animal Medicine, College of Fisheries, Huazhong Agricultural University, Wuhan 430070, China; yangt@tongwei.com (T.Y.); zhaijiale@outlook.com (J.Z.);; 2 Tongwei Agricultural Development Co., Ltd., Tongwei Co., Ltd., Chengdu 610041, China; 3 Division of Fish Disease, Yangtze River Fisheries Research Institute, Chinese Academy of Fishery Sciences, Wuhan 430223, China

**Keywords:** largemouth bass, largemouth bass ranavirus, inactivated vaccine, relative percent survival rates, immune response

## Abstract

The largemouth bass ranavirus (LMBRaV) caused significant mortality and economic loss in the largemouth bass aquaculture industry around the world, including China. Vaccination is an efficient method for virus defense. In this study, an inactivated LMBRaV vaccine was prepared, and the prevention effect as well as the immune responses were analyzed after the primary and the secondary immunization. Compared to the control group, the counts of leucocytes and erythrocytes increased and peaked at day 14 after the primary immunization, and the proportions of leucocytes, including lymphocytes, monocytes, and neutrophils, were also up-regulation after the primary immunization. Serum neutralizing antibody titers increased and peaked (1:128) at day 28 after the primary immunization. Following the secondary immunization, antibody titers were increased to a higher level (1:512) at 28 days after the secondary immunization. Quantitative real-time PCR analysis demonstrated varying degrees of up-regulation of *mhc II*, *igM*, *il-1β*, and *cd8α* transcriptions in the head kidney, which showed that innate and adaptive immune responses were both induced after the primary and the secondary immunization. After challenge with LMBRaV, the relative percent survival rates (RPS) for primary and secondary immunization with inactivated LMBRaV vaccine were determined to be 62.92% and 95.51%, respectively. Therefore, this study suggests that utilizing an inactivated LMBRaV vaccine could induce efficient immune responses and antibody, which might provide a potential efficient countermeasure for LMBRaV prevention.

## Simple Summary

The largemouth bass ranavirus has threatened the largemouth bass industry recently, and vaccination is an efficient method to defend against for virus defense. In this paper, an inactivated largemouth bass ranavirus vaccine was prepared, and both the prevention effect and the immune responses were analyzed after the primary and the secondary immunization. The counts of leucocytes and erythrocytes, the proportions of leucocytes, serum neutralizing antibody titers, and the transcriptions of immune related genes were up-regulated postimmunization. After being challenged with largemouth bass ranavirus, the relative percent survival rates for primary and secondary immunization with inactivated vaccine were 62.92% and 95.51%, respectively. This study that indicated an inactivated largemouth bass ranavirus vaccine could induce efficient innate and adaptive immune responses and antibodies, which might provide a potential efficient countermeasure for disease prevention.

## 1. Introduction

The largemouth bass (*Micropterus salmoides*) belongs to the family *Centrarchidae* and was introduced into China from America [1]. In 2021, its production in China amounted to 802,486 tons, the production is mainly distributed in Guangdong, Zhejiang, Jiangsu, Hunan, Sichuan, and other areas [2]. In recent years, various diseases, including the largemouth bass ranavirus (LMBRaV), have threatened the largemouth bass industry. LMBRaV is also called the Santee-Cooper ranavirus (SCRV) and largemouth bass virus (LMBV). The virus was initially identified in Foshan, Guangdong Province, China, in 2008 and resulted in a significant mortality. Subsequently, LMBRaV pathogens were isolated from diseased fish in Hube, Sichuan, and Jiangsu, among others, and mortality reached 100% [3,4,5].

The largemouth bass ranavirus was initially isolated in Florida in 1991, then spread to 20 states in America as well as Asia. Subsequent analysis of the gene sequences for DNA methyltransferase (DMet) and major capsid protein (MCP) confirmed its taxonomic classification as a member of the *Ranavirus* genus [6,7]. *Ranavirus* infects lower vertebrate species, including fish, amphibians, and reptiles [8]. LMBRaV showed high homology with the ranavirus that infected fish, but it was distinct from the ranavirus that infected amphibians [7]. It has been demonstrated that LMBRaV could infect some species in *Centrarchidae*, such as largemouth bass, smallmouth bass, mandarin fish, spotted bass, blue gill, and others [1]. Developing strategies for LMBRaV prevention are urgent and important for the global aquaculture industry. The infected largemouth bass carry the virus without any symptoms on the body surface; they experience enlargement of the liver and kidneys as well as congestion, but they did not die in a large area [8]. Hence, determining how to control the virus transmission is a big challenge. Although there are some detection methods for LMBRaV [9,10,11,12], there is also a lack of effective treatment methods for virus defense.

Vaccination is considered as a critical strategy for the future defense of aquatic animal diseases. Previously, Ouyang et al. vaccinated grouper with *β*-propiolactone (BPL)-inactivated and formalin-inactivated Singapore grouper iridovirus (SGIV) vaccines, resulting in survival rates of 91.7% and 100% on the 30th day, respectively [13]. Liu et al. found that Chinese giant salamander iridovirus (GSIV) inactivated vaccine showed effective protection against GSIV infection of *Andria davidianus*, with a relative survival rate of 72% after challenge. In addition, the significant increase of *MyD88* and *TLR9* transcriptions confirmed that inactivated vaccine could significantly enhance immune responses [14]. Yi et al. assessed the efficacy of an immersed DNA vaccine against LMBV in largemouth bass. The vaccination demonstrated a relative survival rate of 63% in largemouth bass, and up-regulated immune relative genes in the spleen, head kidney, and liver [15]. Previous studies integrated the major capsid protein of LMBV into the yeast expression system to generate oral vaccines, resulting in survival rates of 66.7% and 41.6, respectively [16,17]. The CotC-LMBV recombinant *Bacillus subtilis* spores demonstrated a relative survival rate of 45% following oral immunization [18]. Although different approaches have been developed to construct LMBRaV vaccines, the safety and commercialization of LMBRaV vaccines still face enormous challenges. In China, the inactivated GCRV vaccine has been commercially available. The safety and operability of the inactivated vaccine has been shown.

In this study, we constructed an inactivated vaccine for LMBRaV and analyzed the protective effect of the vaccine against the LMBRaV infection in largemouth bass, as well as the immunological effects.

## 2. Materials and Methods

### 2.1. Animals

Healthy *Micropterus salmoides* weighing approximately 15 ± 1 g were directly collected from a fish farm located in Hubei province after confirming the absence of LMBRaV through PCR analysis. These fish were acclimated to the laboratory environment for two weeks at a temperature of 28 °C before the experiment and were maintained in a circulating water system and fed daily with commercial food (≥48% protein, ≥6% fat, ≤12% water, and ≤12% ash, Tongwei, Chengdu, China). All the animal handling and experimental procedures were approved by the Animal Care and Use Committee of the Yangtze River Fisheries Research Institute, Chinese Academy of Fishery Sciences (YFI 2024-zhouyong-0425).

### 2.2. Cell Lines and Virus

The epithelioma papulosum cyprini (EPC) cell line, obtained from the China Center for Type Culture Collection (CCTCC), Wuhan University [12], was maintained in medium 199 (M199, Biosharp, Beijing, China) at a temperature of 25 °C, supplemented with 10% fetal bovine serum (FBS, Hyclone, Logan, UT, USA). The LMBRaV-HB001 strain was initially isolated and kept at −80 °C in our lab [5].

### 2.3. Inactivation of LMBRaV

LMBRaV was propagated as previously described [12]. Once the cytopathic effect (CPE) presented about 90%, the virus-containing cells were subjected to three cycles of repeated freezing and thawing. After centrifugation, the virus was stored at −80 °C. For virus titer detection, the LMBRaV was serial diluted 10-fold in the M199 medium, and the 50% tissue culture infectious dose (TCID50/mL) was calculated using the Reed–Muench method as previously described [19]. The formaldehyde was used for LMBRaV inactivation, and the inactivation was performed under 37 °C for 85 h, as described previously [14]. The safety of the inactive vaccine was test by bacterial culture and in vivo study. For the bacterial test, 100 μL of inactive vaccine was aseptically inoculated onto a BHI plate and incubated at 28 °C for 72 h, with no bacterial growth observed. For the in vivo test, ten healthy fish were injected with 100 μL inactive vaccine, and no fish died or showed clinic symptoms in 1 month.

### 2.4. Immunization and Samples Collection

**Figure 1:**
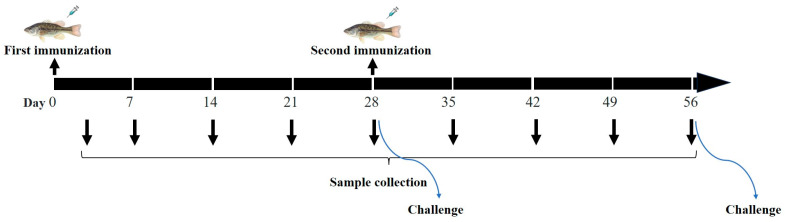
Flowchart outlining the processes for LMBRaV inactivated vaccine immunization.

The healthy *Micropterus salmoides* were divided into control group and immunization group and kept in independent tanks, and each group contained 90 animals. The fish in the immunization group were injected with 100 μL inactivated LMBRaV (1 × 10^6^ TCID_50_ mL^−1^). An equal volume of PBS was injected as control. At day 28 after the primary immunization, 42 immunized largemouth bass were injected with 100 μL inactivated LMBRaV (1 × 10^6^ TCID_50_ mL^−1^) once more, and 30 immunized fish were challenged. Consistently, the same number of control animals were injected with PBS or challenged (Figure 1). The experiments were repeated three times, and each repeat experiment contained one control group (n = 90/repeat) and one immunization group (n = 90/repeat).

Prior to sampling, all the fish were anesthetized with MS-222 (1 mg/mL, pH = 7.2) which was moderate pH with NaHCO_3_. At each time point (1, 3, 7, 14, 21, 28, 35, 42, 49 and 56 days after the primary immunization (dpi), three fish in each tank were collected for blood draw. The collected blood was clotted for 2 h at 4 °C and centrifuged. After centrifuge, the blood was separated to two layers, and the upper layer serum was collected. The head kidney from all above fish were collected and stored in TRIzol Reagent (Invitrogen, Carlsbad, CA, USA).

### 2.5. Blood Parameters

Collected blood was diluted with Dacie’s reagent, and the erythrocytes and leukocytes were counted in a Neubauer chamber using microscopy. For the differential leukocyte count (DLC), the smears were stained with Wright–Giemsa, and 100 leukocytes were randomly counted and classified under microscopy [14]. The experiments were repeated three times, and each sample was tested three times.

### 2.6. Serum Neutralization Test

The sera were treated as previously described [14,17], followed by sequential dilutions prepared using a two-fold reduction (1:2–1:1024) in M199 medium. Each serum dilution was mixed with LMBRaV in equal volumes, followed by incubation at 28 °C for 2 h and seeding into monolayer EPC cells. Then the infected cells were maintained at 28 °C, and CPE was monitored. The highest dilution at which 50% EPC cells were inhibited was considered as the LMBRaV neutralizing antibody titer. The experiments repeated three times, and each sample was tested three times.

### 2.7. Quantitative Real-Time Reverse Transcriptase PCR (qRT-PCR)

The total RNA of the head kidney of largemouth bass was extracted using Trizol reagent (Invitrogen, Carlsbad, CA, USA), and then the genomic DNA was digested by DNase [20]. cDNA was synthesized using the ReverlAid First Stand cDNA Synthesis Kit (Thermo Scientific, Waltham, MA, USA) and stored at −20 °C. The specific primers were used for quantification expression (Table 1). Quantitative real-time PCR was performed using the Hieff qPCR SYBR Green Master Mix (Yesen, Shanghai, China). qRT-PCR was performed with 95 °C for 30 s, 95 °C for 15 s, 60 °C for 20 s, and 72 °C for 35 s; steps 2–4 were repeated for 40 cycles. The experiments were repeated three times, and each sample was tested three times.

### 2.8. LMBRaV Challenge

To assess the protective efficacy of LMBRaV vaccine in *Micropterus salmoides*, intraperitoneal injections of 100 μL of live LMBRaV virus (1.0 × 10^6^ TCID_50 mL_^−1^) were administered at day 28 and 56 after the primary immunization, respectively, with a total of 30 fish in each experimental group. The mortality rate was monitored for a duration of two weeks, and the dead fish were removed in a timely fashion. The waste water was disinfected by povidone iodine. The relative percent survival (RPS) was determined using the formula proposed by Amend in 1981 [22]. The experiments repeated three times.

### 2.9. Statistical Analysis

Three replicates of each sample were expressed as means (±SD). The statistical analysis was assessed using one-way ANOVA and Duncan multiple range test in the GraphPad Prism 8.0. For mortality curves analysis, Kaplan–Meier method and Mantel–Cox test was used, and *p* ≤ 0.05 was considered to be significant.

## 3. Results

### 3.1. Blood Cell Counting

**Figure 2:**
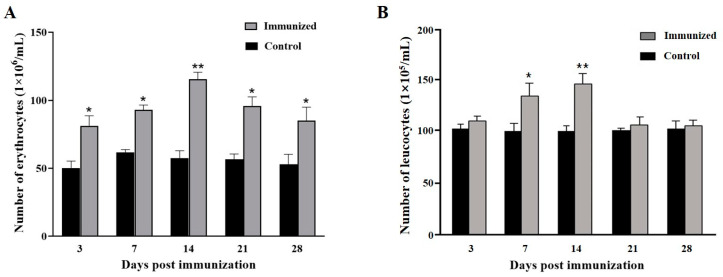
Changes in the numbers of erythrocytes (**A**) and leucocytes (**B**) in the peripheral blood of *Micropterus salmoides* immunized with inactivated LMBRaV. Three replicates were set for the tests, with three fish per replicate. Data are presented as mean SD. * *p* ≤ 0.05; ** *p* ≤ 0.01.

Compared to the control group, the number of erythrocytes of the immunized group increased at day 3 after the primary immunization, peaked at day 14 ((115 ± 6) × 10^6^/mL), then decreased, but remained higher than those of the control group. In the control group, the number of erythrocytes were maintained at (50 ± 5) × 10^6^/mL (Figure 2A). The number of leucocytes increased at day 7 after the primary immunization and reached a peak at day 14 ((146 ± 9) × 10^5^/mL) in the immunized group. In the control group, the number of leucocytes were maintained at about (100 ± 5) × 10^5^/mL (Figure 2B).

### 3.2. Different Leucocytes Count

**Figure 3:**
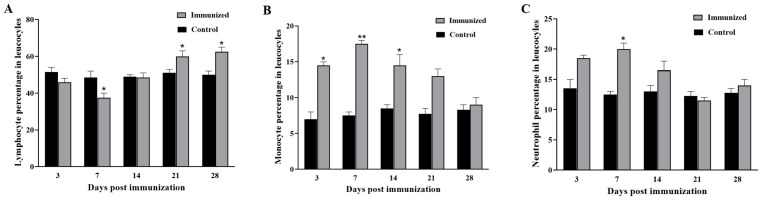
Changes in the differential leukocyte counts of lymphocytes (**A**), monocyte (**B**) and neutrophil (**C**) in the peripheral blood of *Micropterus salmoides* immunized with inactivated LMBRaV. Three replicates were set for the tests, with three fish per replicate. Data are presented as mean SD. * *p* ≤ 0.05; ** *p* ≤ 0.01.

After the primary immunization, the percentage of lymphocytes were decreased at the first week after immunization, then increased to a peak at day 21 and were maintained at high level (Figure 3A). The percentage of monocyte was significantly increased at day 3 and peaked at day 7 after the primary immunization, then decreased to the control level at day 28 (Figure 3B). The percentage of neutrophil increased at day 3, reached a peak at day 7, then decreased to the control level at day 21 after the primary immunization (Figure 3C).

### 3.3. Serum Antibody Levels

**Figure 4:**
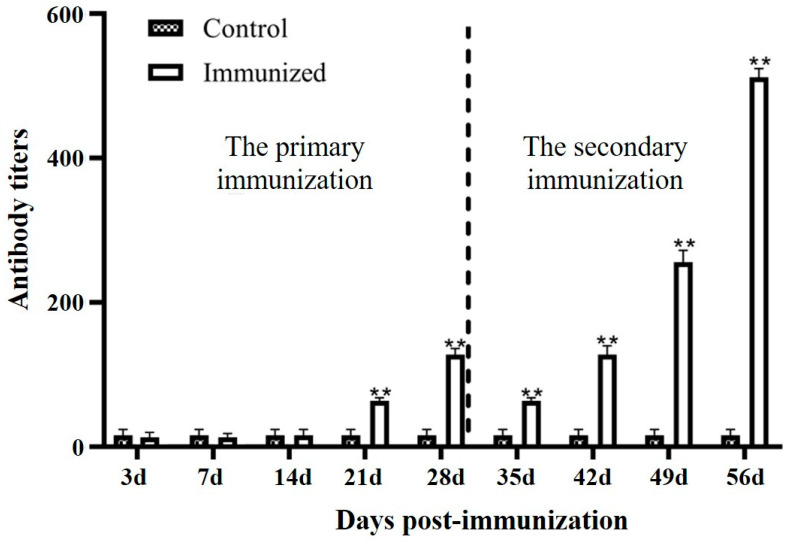
Serum antibody titers of *Micropterus salmoides* immunized with inactivated LMBRaV. The neutralization titers were expressed as reciprocal values of the sera dilution given without CPE in the well with EPC cells inoculated with LMBRaV. Three replicates were set for the tests, with three fish per replicate. Data are presented as mean SD. ** *p* ≤ 0.01.

The viral neutralization analysis revealed that the serum antibody levels in the vaccinated group significantly increased at day 21 after the primary immunization, contrasting with the control group. The neutralizing antibody titer (1:128) was observed at day 28 after the primary immunization. In addition, significantly increased antibody titers were detected after the second immunization. The highest neutralizing antibody titer (1:512) was achieved at day 28 after the secondary immunization (56 days after the primary immunization) (Figure 4).

### 3.4. Transcription Changes of Immune Related Genes

**Figure 5:**
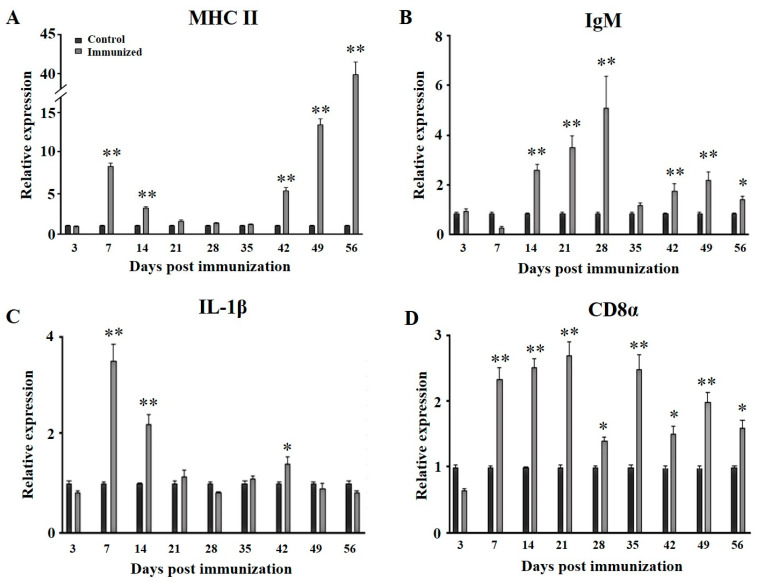
qRT-PCR analysis of the expression levels of immune-related genes of the head kidney postvaccination. (**A**): Expressions of *mhcⅡ* in the head kidney, (**B**): expressions of *IgM* in the head kidney, (**C**): expressions of *IL1β* in the head kidney, (**D**): expressions of *CD8*α in the head kidney. The mRNA level of each gene was normalized in reference to the expression of the *β-actin* gene. For each gene, the mRNA level of the control animals was set as 1. Three replicates were set for the tests, with three fish per replicate. Data are presented as mean SD. * *p* ≤ 0.05; ** *p* ≤ 0.01.

To evaluate the immune responses after immunization, the transcription levels of *mhc II*, *igM, il-1β*, and *cd8α* were tested using qRT-PCR. In the head kidney, *mhc II* transcription levels increased at day 7 after the primary immunization, and were then down-regulated to the same level as the control group. After the secondary immunization, the transcriptions were up-regulated again and reached the highest level at day 28 (day 56 after the primary immunization) (Figure 5A). The transcription levels of *igM* were up-regulated from day 14 to day 28 after the primary immunization. During the secondary immunization, the *igM* transcription levels were up-regulated to the highest level at 21 days after the secondary immunization (day 42 and day 49 after the primary immunization) (Figure 5B). The *il-1β* transcriptions in the head kidney were up-regulated at 7 and 14 days after the primary immunization, and then down-regulated to the control level. After the secondary immunization, the transcriptions were triggered again at day 14 (day 42 after the primary immunization) (Figure 5C). The transcription levels of *cd8α* were up-regulated from day 7 to day 21 after the primary immunization and then down-regulated, the transcriptions were up-regulated again at day 7 after the secondary immunization (day 35 after the primary immunization) and maintained at a high level (Figure 5D).

### 3.5. Challenge Test

**Figure 6:**
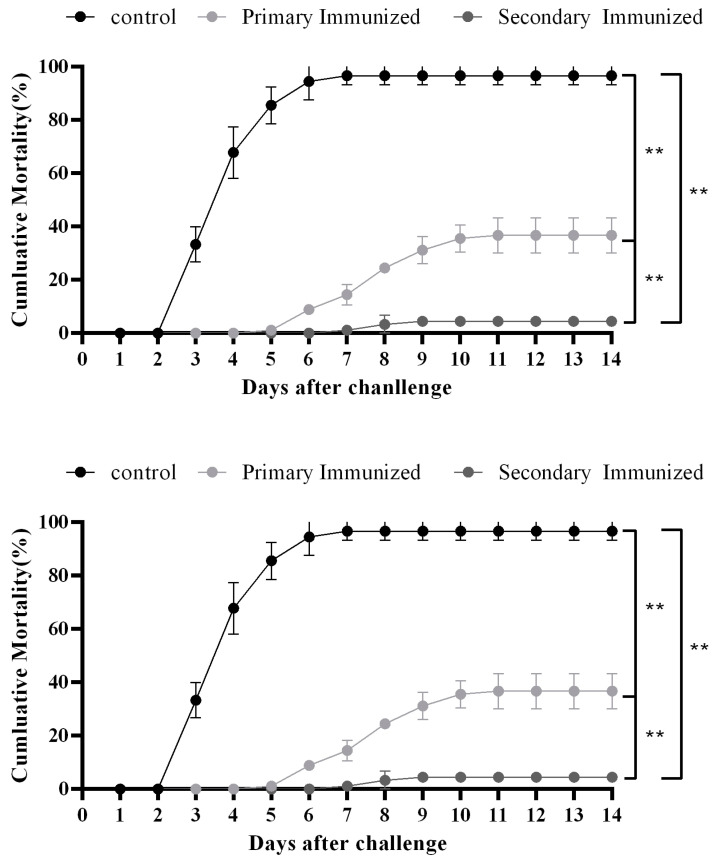
Cumulative mortality curves of immunized and control *Micropterus salmoides* after LMBRaV infection. Three replicates were set for the tests. ** *p* ≤ 0.01.

Postinfection with the LMBRaV, the control fish began to die at 2 days after infection, and the cumulative mortality reached 96.67% at day 14 postinfection. The mortality of primary and secondary immunization groups began at 6 and 8 days after infection, respectively, meanwhile the cumulative mortality significantly decreased to 4.44% and 36.67%, respectively. The RPS of the inactivated LMBRaV vaccine was 62.92% and 95.51% after the primary and secondary immunization, respectively (Figure 6).

## 4. Discussion

In fish, the stimulation of innate and adaptive immune response by vaccine results in the production of specific antibodies, which enhances the defense capability [23]. Inactivated vaccines have been widely used in aquatic animals and present a high protection effect. The *Andria davidianus* immunized with the inactivated GSIV exhibited high antibody titer and high protection effect [14]. Both BPL-inactivated and formalin-inactivated SGIV vaccines induced immune response in grouper and showed high survival rates (91.7% and 100%, respectively) [13]. Zhang et al. prepared an inactivated CyHV-2 vaccine and investigated its protective mechanism in gibel carp. The results indicated that the percentage of phagocytes and phagocytic were induced, and the serum neutralizing antibody titer increased to peak at 21 days postimmunization. Furthermore, the immunized fish exhibited a higher RPS (71.4%) [24]. Luo et al. reported formalin inactivated LMBV-2007064 vaccine, and the RPS was 25% [25]. The properties of the virus strain were critical elements in determining the effect of inactivated virus vaccines [26]. Hence, in this study, we vaccinated largemouth bass with inactivated LMBRaV-HB001 vaccine, and the immune responses and protection effect were evaluated.

The health and immune status of aquatilia could be assessed through hematology [27]. Wang et al. reported that the erythrocytes of amphibians might have immune defense functions [28]. Additionally, phagocytic activity of erythrocytes was detected in crustaceans, amphibians, and fish, which indicated that the erythrocytes played an important role in the non-specific immune responses [14]. Moreover, the erythrocyte number increased after vaccine immunization in amphibians and fish [14,24,28]. In this research, the number of erythrocytes increased and peaked at 14 days after immunization, which might indicate the non-specific immune responses activation postimmunization. After the primary immunization, the proportion of lymphocytes increased significantly at day 21, while the monocytes and neutrophils reached highest levels at day 7. These results revealed that the lymphocytes activation might be later than the monocytes and neutrophils activations, which was similar to inactivated GSIV and CyHV-2 vaccine [14,24].

The presence of neutralizing antibodies in fish after vaccination can serve as an indicator of their ability to effectively combat viral infection [29]. The neutralization titer of the LMBV DNA vaccine reached a maximum of 1:375 on day 14 postimmunization, and the RPS was 63% [15]. Yao et al. prepared the LMBV *Pichia pastoris* oral vaccine, which showed the highest antibody level on 28 days after immunization, and the RPS was 41.6% [16]. Zhang et al. constructed the LMBV *Saccharomyces cerevisiae* oral vaccine which displayed MCP protein and the LTB protein as an adjuvant, and the maximum antibody titer (1:85) was reached at day 28, and the RPS was 66.7% [17]. Wang et al. developed a *Bacillus subtilis* spores with LMBV MCP displayed on the surface, which induced abundant specific antibodies and reduced viral load, and the RPS was 45% [18]. In this study, the inactivated LMBRaV vaccine induced an antibody titer of 1:512 and high RPS (95.51%) after the secondary immunization, which had a higher protective efficacy than the DNA vaccine and oral vaccines. These results might be due to the vaccination approach, immunization frequency, and antigen type. Although the LMBV egg yolk antibody had been produced by immunization in the LMBV inactivated vaccine and showed high antibody titer, the in vivo protection effect of the egg yolk antibody was still unknown [30]. Moreover, the RPS of the secondary immunization (95.51%) was higher than that of the primary immunization (62.92%). In mammals, the secondary immune response induced by booster vaccination stimulates rapid, accelerated, and increased antibody production [31]. In teleost, the booster vaccination of CyHV-2 inactivated vaccine demonstrated that the RPS increased to 63.6% in the secondary vaccination group, while it was 42.5% in the primary vaccination group [32]. The results of our study also demonstrated a notable enhancement of antibody titers as well as the survival rate following the secondary immunization. Although the booster immunization came with higher costs and complex operation, the significantly higher RPS reduced the enormous economic loss, which made it worthy of application. In addition, the protection duration of the secondary vaccination needs to be tested in future. As previously reported, the RPS of LMBV-2007064 inactive vaccine without adjuvant were 25% and 31.25%, respectively [25], which suggests that the LMBRaV-HB001 strain might be a better candidate virus strain for inactive vaccine development.

To further assess the immunologic effects of the vaccine, our study focused on the immune-related genes, such as *mhc II*, *igM*, *il-1β*, and *cd8α*, in the head kidney. As is well known, the head kidney is the critical immune organ in fish [33]. The MHC molecules are primarily expressed on the surface of the antigen-presenting cells (APCs). The APCs phagocytose and catabolize the pathogens into small peptides and subsequently bind to MHC class II molecules, and finally form peptide-MHC class II complexes. These complexes then interact with CD4 + T cell surface receptors (TCR) to form trimeric complexes, which activate CD4 + T cells involved in humoral immune responses and cellular immune responses. CD8 + T cells are the other type of T cell, which involve in cellular immune responses [34,35,36]. CD8α is a surface receptor of CD8 + T cells, and the gene up-regulation was considered as a marker of active cytotoxic T cells [37]. In our study, the expressions of *mhc II* and *cd8α* both exhibited a peak on day 7 following the primary immunization. Consistently, significant up-regulation of *mhc II* and *cd8* expressions were detected in largemouth bass immunized with the LMBV *Pichia pastoris* oral vaccine [16]. These results suggested that LMBRaV inactivated vaccine might induce antigen presentation which subsequently elicit adaptive immunity. The IgM, a subclass of immunoglobulins, plays a pivotal part in the teleost humoral immunity, and is regarded as the primary antibody [38]. The recombinant oral LMBV vaccines and subunit vaccine all induced notable increased *igM* expressions [16,17,38]. In this study, the expressions of *igM* were also significantly increased after immunization with the inactivated vaccine, which is consistent with the inducible antibody. The inflammatory response is an efficient anti-virus response in innate immune of vertebrates. The inflammatory cytokine IL-1β triggers inflammatory responses following immunization with LMBV DNA vaccine and recombinant oral vaccine [15,17]. In the present study, the up-regulation of *il-β* expressions were also observed from 7 to 14 days after immunization, suggesting the involvement of the inflammatory response during this specific period in largemouth bass. Meanwhile, we noticed that the transcriptions of *mhc II*, *igM*, *il-1β* and *cd8α* were all up-regulated again after the secondary immunization, which might imply that the vaccine induces innate and adaptive immune responses one more time. Compared to the primary immunization, the high transcription level of *mhc II* after the secondary immunization lasted longer and reached a higher level, which might indicate the long-term and potent antigen presentation of the secondary immunization. In rainbow trout that were immersion vaccinated three times, the density of macrophages (an important APCs) was increased [39], which might be related to enhanced antigen presentation. These results might be related to the rapid and enhanced antibody production and higher antiviral activity of the booster vaccination.

## 5. Conclusions

In summary, the LMBRaV-HB001 inactivated vaccine demonstrated significant efficacy in providing protection against LMBRaV infection of largemouth bass. Notably, the antibody titer displayed a marked increase, and the immune responses were induced after immunizations. These findings suggested that the LMBRaV-HB001 inactivated vaccine holds promise as a potential strategy for combating LMBRaV virus. Further studies are warranted to investigate its long-term protective effects and more convenient immunization methods.

## Figures and Tables

**Table 1:** PCR primers used in present study.

Gene	Sequence (5′–3′)	Accession no. or Reference	Usage
*il-1* *β*	CCGTGCCAACAGTGTGAAGA	XM_038733429	qRT-PCR
	GGGTGCTGTGTCCACCTTGC		
*igM*	CGACTACGATATGAACTGGG	XM_038698199	qRT-PCR
	GCTGTTGTCTCTGGAGATGG		
*mhc II*	GGGATGGAGACCAGGCGATA	XM_038735684 XM_038696403	qRT-PCR qRT-PCR
*cd8* *α*	CCCGCTTGACAGCACATCCT CCAAGTCAGTGCACATCTAC GGGCCCAGTATGATTGAAGG		
*β-actin*	CCACCACAGCCGAGAGGGAA	[21]	qRT-PCR
	TCATGGTGGATGGGGCCAGG		

## Data Availability

The original contributions presented in the study are included in the article, further inquiries can be directed to the corresponding authors.
